# Elizabeth June Horder 1920–2017

**DOI:** 10.1080/17571472.2017.1339501

**Published:** 2017-06-15

**Authors:** Paul Thomas, Hugh Barr, Gillian Yudkin

Elizabeth to her colleagues; June to her family and friends; she touched the lives of so many with her gentleness and piercing intellect. She was the eldest of three daughters of Maurice and Dorothy Wilson, living in Stocksfield and Northumberland. Following the early death of her father, the family moved to Switzerland, Edinburgh, then Oxford. She met John Horder while studying in Paris and their relationship began in Oxford where they were both students. They married in 1940, when she was nineteen.

June was a model team-player, always there when the situation required, then melting into the background when things were under control. She would notice everything and everyone, remember your personal details, enquire about the needs of others, and quietly work ‘offstage’ to help you to perform well. She was always generous in her hospitality and made you feel that you really mattered. She had a great love of music and gardening and this showed in the way she carried herself – in harmony with the world. She enjoyed lively and challenging conversation, and spoke with humour and wisdom. You could not help but listen to every word, and then fall under her spell. And remember her with great affection.

She was an inspirational trainer, demonstrating care, compassion, conscientiousness and personalised care. She earned the love of all her patients from whatever stratum of society they came. She was an early exponent of multidisciplinary team-working, totally valuing the roles of health visitors, geriatric visitors and social workers as well as encouraging practice nurses before others had even dreamt of them.

There were two Doctor Horders. John, June’s husband, is internationally known as the father of modern-day broad-visioned primary care. June matched John in every way. They were the best team imaginable. As well as shaping healthcare history in their 72 years of life together, they brought up four children, seven grandchildren and two great grandchildren. Together they inspired a generation.

Reflecting in this Journal [1] on her career in General Practice, Elizabeth recalled being appointed in 1946 as Assistant GP to Dr. James Wigg in Kentish Town to augment his singleton practice working with a ‘panel’ of about 2000 insured patients from his home. She was an unwavering supporter of the NHS, as well as a caring and compassionate doctor. With like-minded colleagues she developed a multi-professional team to care for a socially and racially diverse population, while maintaining, as far as was possible, personal lists of patients. You can read more about Elizabeth and those pioneering days in John’s autobiography, serialised in this journal [2].

Elizabeth June was exciting, caring, inspiring and beautiful to the end. She referred rarely to her contribution to healthcare evolution; still less, mindful of its sensitivity, to her longstanding voluntary work in ‘retirement’ with Child Line and Victims of Torture.

We miss you.

A memorial to celebrate her memory will be held on Saturday, 22 July 3 to 6 pm at St Mary’s Church, Primrose Hill, Elsworthy Road, London NW3 3DJ.

Thanks to William, Tim and Josephine Horder, Annabelle Sorge, and Peter Williams for guidance in writing this obituary.
